# Two new species of *Oreocharis* (Gesneriaceae) from karst regions in Yunnan and notes on *O. tetraptera* and *O. brachypoda* from China

**DOI:** 10.3897/phytokeys.162.52174

**Published:** 2020-10-07

**Authors:** Lei Cai, Zhang-Jie Huang, Fang Wen, Zhi-Ling Dao

**Affiliations:** 1 Yunnan Key Laboratory for Integrative Conservation of Plant Species with Extremely Small Populations, and Key Laboratory for Plant Diversity and Biogeography of East Asia, Kunming Institute of Botany, Chinese Academy of Sciences, Kunming 650201, Yunnan, China Kunming Institute of Botany, Chinese Academy of Sciences Kunming China; 2 Guangxi Key Laboratory of Plant Conservation and Restoration Ecology in Karst Terrain, Guangxi Institute of Botany, Guangxi Zhuang Autonomous Region and Chinese Academy of Sciences, Guilin 541006, Guangxi, China Guangxi Institute of Botany, Guangxi Zhuang Autonomous Region and Chinese Academy of Sciences Guilin China; 3 Gesneriad Conservation Center of China, Guilin Botanical Garden, Chinese Academy of Sciences, Guilin 541006, Guangxi, China Guilin Botanical Garden, Chinese Academy of Sciences Guilin China

**Keywords:** flora of Yunnan, limestone area, morphology, new taxon, *
Oreocharis
*

## Abstract

Two new species of Gesneriaceae, *Oreocharis aimodisca* and *O. longipedicellata*, from the limestone area of Yunnan Province, China, are described and illustrated. Their morphological relationship with similar species is discussed and colour photographs, detailed descriptions, distribution and habitat, as well as the IUCN endangered status are provided. We also discuss the accuracy of the scientific names of the described species *O. tetrapterus* from Guangxi, China in 2019 and *O. brachypodus* from Guizhou, China, in 2015, and put forward corrections related to name form.

## Introduction

At least 30 new taxa of *Oreocharis* Benth. (Gesneriaceae) have been described and officially published (e.g., Cai et al. 2017, 2019; Do et al. 2017; Chen et al. 2018; Guo et al. 2018; Möller et al. 2018; Pan et al. 2019; Yang et al. 2019) after the generic redefinition based on new evidence following the development of molecular phylogenetics in 2011 (Möller et al. 2011) and several later adjustments of the species (Middleton et al. 2013; Chen et al. 2014; Möller et al. 2014; Möller 2015). *Oreochariss.l.* hitherto comprises more than 140 taxa, mainly distributed in South and Southwest China (Wen et al. 2014, 2019) and a few species extending into North Vietnam (nine species), Myanmar (two species), Bhutan (one species), India (one species), Japan (one species) and Thailand (one species) (Xuyen et al. 2016; Do et al. 2017; Möller et al. 2017, 2018; Chen et al. 2018).

Li and Li (2015) and Pan et al. (2019) each described new species of *Oreocharis*. One of the taxa with four corolla lobes is from Guangxi, China, which they named *O. tetrapterus* F.Wen, B.Pan & T.V.Do (Pan et al. 2019). The other has sessile or shorter petiole leaves and four stamens with anthers coherent in pairs from Guizhou, China, which was named as *O. brachypodus* J.M. Li & Z.M. Li (Li and Li 2015). These scientific names are improperly formed because the Latin forms ‘*tetrapterous*’ and ‘*brachypodus*’ are masculine and the Latin word of this genus, ‘*Oreocharis*’, is feminine. We revise the Latin name to ‘*tetraptera*’ and ‘*brachypoda*’ here and provide appropriate notes.

In 2018, during field investigations in the limestone area in Southeast Yunnan, China, an unknown species of Gesneriaceae without flowers was collected, then was introduced to, and cultivated in, Guilin Botanical Garden (GBG). We first observed flowering plants which were cultivated in GBG in August 2019. Thereafter, in September 2019, another unknown species of Gesneriaceae with flowers was collected from Shizong County, eastern Yunnan. We confirmed that they are both members of *Oreocharis*, based on the characteristics of leaves in a basal rosette, four separated fertile stamens and capsules dehiscing predominantly on one side. After a careful examination of the related specimens and taxonomic publications of *Oreocharis* from the adjacent regions (Wang et al. 1990, 1998; Li and Wang 2005), we concluded that these two species are both new to science. Here, *Oreocharis aimodisca* and *O. longipedicellata* are described and illustrated and their morphological characters are compared to closely-related species.

## Material and methods

Extensive fieldwork has been undertaken in the east and southeast of Yunnan, China, in recent years. Samples of the two new species were respectively collected from the fields of Shizong County and living plants cultivated in Guilin Botanical Garden (GBG) which initially introduced from Malipo County, Yunnan, China. All available specimens of *Oreochariss.l.*, stored in herbaria (E, HITBC, IBK, HN, K, KUN, P, PE and VMN), Chinese Virtual Herbarium (http://www.cvh.ac.cn/) in China and Global Plants on JSTOR (https://plants.jstor.org/) were examined. We studied all morphological characters with dissecting microscopes and described the morphological characters by using the terminology presented by Wang et al. (1990, 1998). The photographs and the specimens were taken in the field and GBG by the first and correspondence authors. All specimens seen are indicated by ‘!’.

## Taxonomic treatment

### Oreocharis tetraptera

Taxon classificationPlantaeLamialesGesneriaceae

F.Wen, B.Pan & T.V.Do

544D61EE-8729-5929-AFB4-57971A74DFBD

#### Orthographic variant.

*Oreocharis tetrapterus* F.Wen, B.Pan & T.V.Do in Pan et al. 2019: 83.

#### Type.

China. Guangxi: Hezhou City, Lisong Town, Gupo Mountain, 24°39'N, 111°36'E, elev. ca. 950 m, on moist surface of granite rocks, in flower, 25 August 2018, Wen Fang WF160825-01 (holotype: IBK!, isotype: IBK!).

### Oreocharis brachypoda

Taxon classificationPlantaeLamialesGesneriaceae

J.M. Li & Z.M. Li

C58DA02D-B175-5420-A6CB-B69914A1B281

#### Orthographic variant.

*Oreocharis brachypodus* J.M. Li & Z.M. Li in Li and Li 2015: 296.

#### Type.

China. Guizhou: in the vicinity of Tongren city, on rather cool rocks and very steep banks of cool, clammy soil that grows a fine film of moss, elev. 1300 m, 9 April 2014, *Jia-Mei Li 2304* (holotype: HEAC!); ibid. *Jia-Mei Li 2305* (paratype: HEAC!).

#### Notes.

The gender of the genus name, *Oreocharis*, is feminine, but the suffix of the scientific name, “-*us*,” is typically masculine. For *Oreocharis tetrapterus* (Pan et al. 2019), the correct orthography of the name of the new species is *O. tetraptera*, is written by using an inaccurate gender, namely “*tetrapterus*”, in the citation of the type of the new species (p. 85), in the discussion of the Etymology (p. 86) and in the notes of the illustration (pp. 86, 87 and 88). In the other new taxon, *Oreocharis brachypodus* (Li and Li 2015), the correct orthography of the epithet “*brachypoda*” should be used to replace “*brachypodus*”. The inaccurately-used name gender appeared in the citation of the type of the new species (p. 296) and in the notes of the illustration (pp. 297 and 298). Thus, here we correct and revise two new species’ names as *Oreocharis tetraptera* and *O. brachypoda*.

### Oreocharis aimodisca

Taxon classificationPlantaeLamialesGesneriaceae

Lei Cai, Z.L.Dao & F.Wen sp. nov.

B2A5E9EE-853D-545C-AECA-93278A5D507E

urn:lsid:ipni.org:names:77211926-1

Figures 1 , 2 , 3

#### Diagnosis.

*Oreocharis aimodisca* is morphologically similar to *O. longifolia* (Craib) Mich.Möller & A.Weber and *O. muscicola* (Diels) Mich.Möller & A.Weber in the appearance and colour of its flowers, but differs from the latter two species in its leaf blade oval to ovate, base cordate or auriculate, margin crenate, peduncle densely brown villous and pubescent, corolla outside densely pubescent and four separated fertile stamens, pistil densely pubescent and disc blood red.

**Figure 1.**
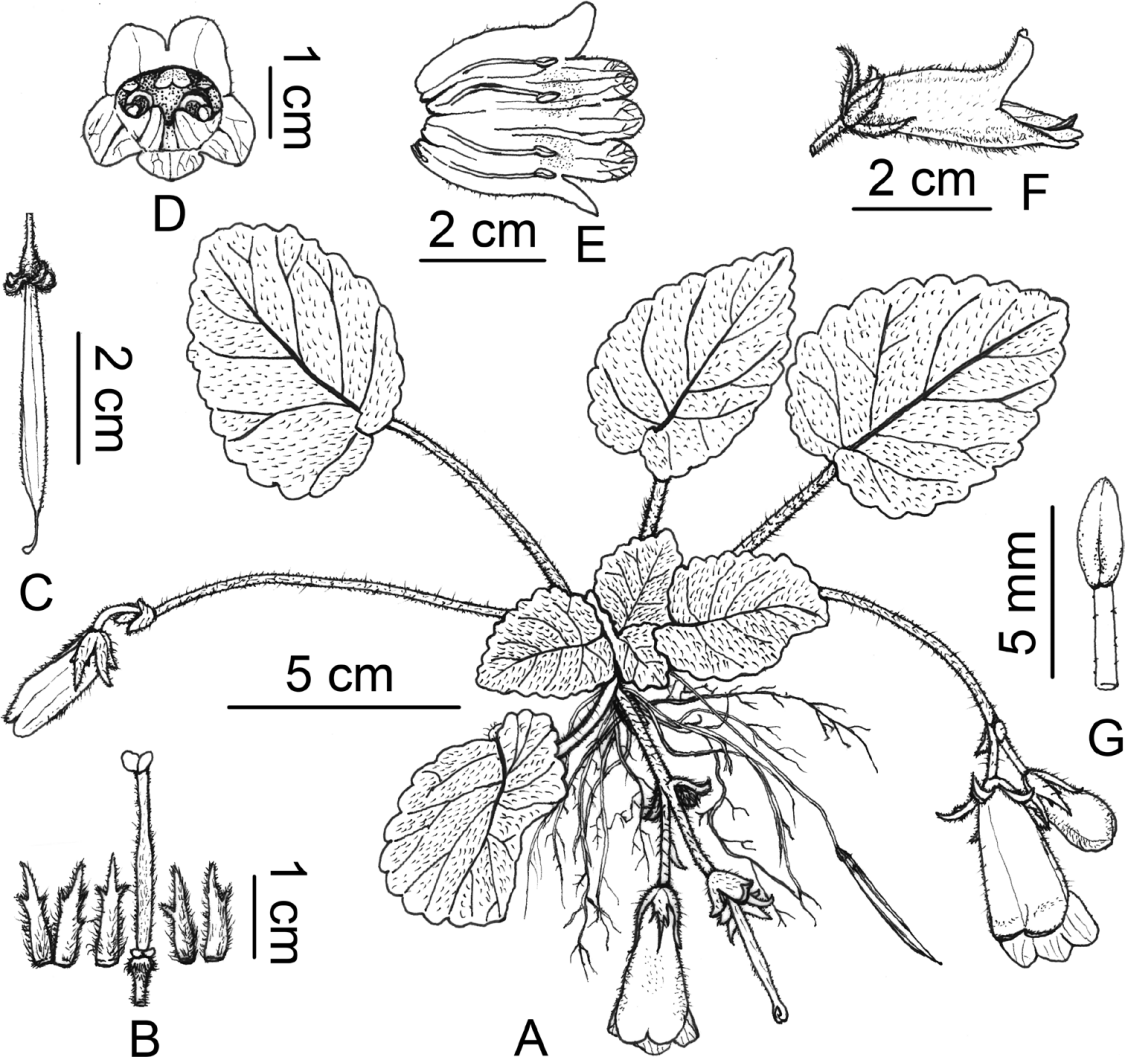
*Oreocharis aimodisca* Lei Cai, Z.L.Dao & F.Wen, sp. nov. **A** habit **B** pistil with disc and calyx **C** old capsule **D** front view of flower **E** opened corolla showing stamens and staminode **F** side view of a flower **G** adaxial view of the anther. Drawn by Xuan-Lin Zhu.

#### Type.

China. Yunnan: Shizong County, Wulong Town, Dachang Village, Xiaofakuai, 24°39'N, 104°10'E, elev. ca. 2122 m, on the surface of wet rocks, in flower, 10 September 2019, *Lei Cai & Pin Zhang CL275* (holotype: KUN!, isotypes: KUN!, IBK!).

#### Description.

Perennial herb, rhizome 5–18 mm long, 3–5 mm in diameter. Leaves 6–18, basal, petiole 2.5–10.5 cm long, brown villous and pubescent, leaf blade oval to ovate, 2.5–7.0 × 1.8–5.5 cm, adaxially densely appressed pubescent, abaxially puberulent, densely brown pubescent along veins, lateral veins 3–6 on each side of midrib, adaxially inconspicuous, abaxially conspicuous, apex acute, base cordate or auriculate, slightly oblique sometimes, margin crenate. Cymes axillary 2–5, inflorescence 1–5-flowered; peduncle 5.5–16 cm long, brown villous and pubescent; bracts 2, lanceolate to narrowly triangle, 5–8 × 1.5–2.8 mm, outside brown pubescent, inside glabrous, margin nearly entire to denticulate; pedicel 1.2–3.5 cm long, densely pubescent. Calyx 8–12 mm long, 5-lobed to the base, lobes unequal, linear-lanceolate or narrowly triangular, 8–12 × 1.5–2.2 mm, both sides densely pubescent, margin denticulate. Corolla yellow, 2.8–3.6 cm long, outside densely pubescent, inside puberulent in the throat and on adaxial lobes, the lower part forms red stripes on the throat and lobes, tube coarsely tubular, gradually expanded from base to the throat, 2.0–2.6 cm long, 6–10 mm in diameter; limb 2-lipped; adaxial lip 2-lobed to middle, semicircular, lobes 4–5 × 4–5 mm, abaxial lip 3-lobed to middle, semicircular, 5–6 × 5–7 mm. Stamens 4, 1.5–1.8 cm long, adnate to corolla 4–7 mm from the base; filaments linear, sparsely pubescent; anthers oblong, 2-loculed, dehiscing longitudinally, connective glabrous; staminode 1, 0.6–0.8 mm long, inserted ca. 3 mm from the base. Disc ca. 1.2 mm high, blood red, margin undulate. Pistil 1.6–2.4 cm long; ovary long cylindrical, densely pubescent, 1.0–1.4 cm long; style 6–10 mm long, densely pubescent; stigma bilobed, flabellate. Capsule linear, 3.5–4.8 cm long.

**Figure 2.**
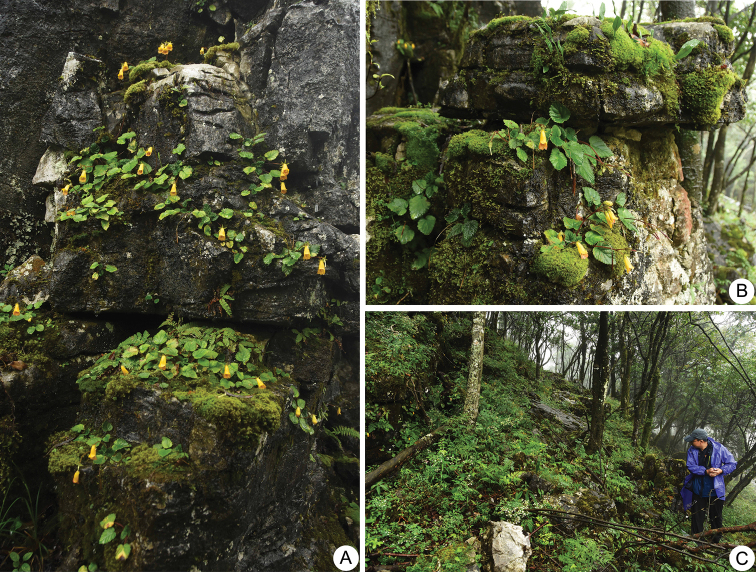
*Oreocharis aimodisca* Lei Cai, Z.L.Dao & F.Wen, sp. nov. in natural habitat **A, B** plants with flowers in the wild **C** habitat. Photographed by Lei Cai.

#### Phenology.

Flowering from August to September; fruiting from September to December.

#### Distribution and habitat.

*Oreocharis aimodisca* is currently known from two adjacent populations at the type locality, Shizong County, East Yunnan, China. The new species commonly growing with other plants in shady and wet places on the middle part of mountain slopes under primary evergreen broad-leaf forest and shrubbery on karstic limestone at an elevation of over 2000 m.

#### Etymology.

The original specific epithet ‘*aimodisca*’ derives from the Greek ‘αίμα’ meaning blood red and ‘δίσκος’ meaning discus.

#### Vernacular name.

The Chinese name of the new species is “Diān Dōng Mǎ Líng Jù Tái” (滇东马铃苣苔). The first two words, “Diān Dōng,” mean east of Yunnan, the next four words, “Mǎ Líng Jù Tái,” mean *Oreocharis* in Mandarin.

#### Conservation status.

Based on our field investigations, the new species is currently only known from the type locality with two contiguous subpopulations, in total ca. one thousand mature individuals were present within 5000 m^2^ (AOO). Since no special surveys were carried out for delimiting its distribution and information about threats is not very clear, this species was provisionally considered to be Critically Endangered [CR B2(a)] in terms of the IUCN Red List Categories and Criteria (IUCN 2019).

#### Taxonomic affinities.

*Oreocharis aimodisca* is morphologically similar to *O. longifolia* and *O. muscicola* in the corolla yellow and coarsely tubular; however, it is different from the latter two species by the shape of the leaf blade, indumentum characters of the peduncle, pedicel, calyx, corolla and pistil and separated stamens. The comparison of morphological characters on related species is provided in Table 1.

**Figure 3.**
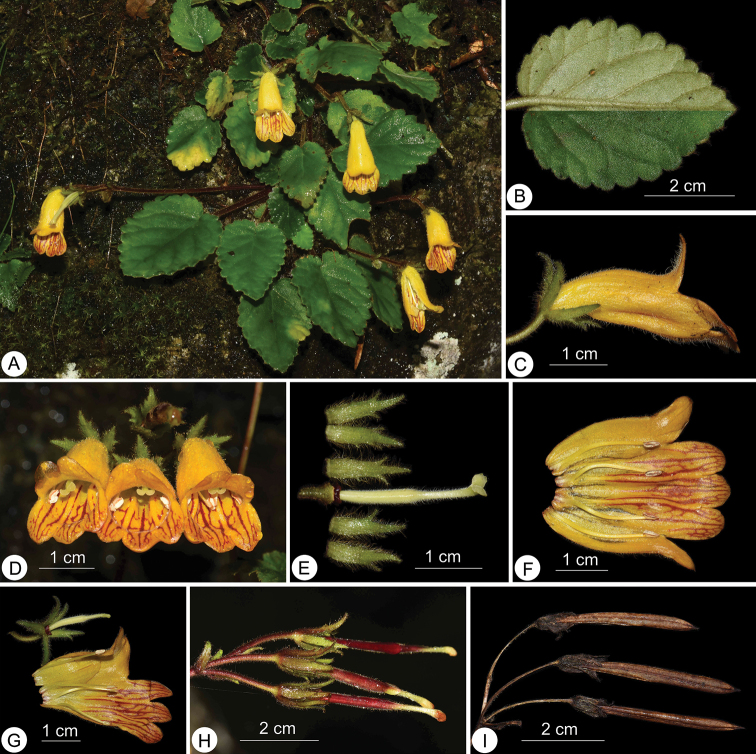
*Oreocharis aimodisca* Lei Cai, Z.L.Dao & F.Wen, sp. nov. **A** habit **B** adaxial (lower half) and abaxial leaf surface (upper half) **C** side view of a flower **D** front view of flowers **E** pistil with disc and calyx **F** opened corolla showing stamens and staminode **G** opened corolla with pistil and calyx **H** fresh fruits **I** old capsules. Photographed by Lei Cai.

**Table 1.** Morphological comparison of *Oreocharis aimodisca* sp. nov., *O. longifolia* and *O. muscicola*.

Characters	*O. aimodisca*	*O. longifolia*	*O. muscicola*
**Leaf-blade**	oval to ovate, base cordate or auriculate, margin crenate	narrowly elliptic to oblanceolate, base attenuate, margin serrulate	narrowly elliptic to lanceolate, base often slightly oblique, narrowly to broadly cuneate, margin serrate to serrate-crenate, sometimes doubly so
**Petiole**	brown villous and pubescent	grey to brownish pubescent	densely rust-brown villous
**Peduncle**	densely brown villous and pubescent	sparsely brownish villous to pubescent	rust-brown villous and glandular-pubescent
**Bract**	lanceolate to narrowly triangle, outside brown pubescent margin nearly entire to denticulate	oblanceolate, outside pubescent, margin entire	lanceolate, outside rust-brown villous, margin entire
**Calyx**	lobes linear-lanceolate or narrowly triangular, both sides densely pubescent, margin denticulate	narrowly ovate, outside sparsely brownish pubescent, inside glabrous, margin entire	lanceolate, outside sparsely white pubescent and rust-brown villous, margin entire
**Corolla**	outside densely pubescent, inside puberulent in the throat and on adaxial lobes	outside sparsely glandular puberulent, inside sparsely glandular puberulent	outside sparsely puberulent, inside glandular pubescent
**Filaments**	sparsely pubescent	glabrous	sparsely puberulent
**Anthers**	oblong, separated	reniform, connected in pairs	reniform, connected in pairs
**Pistil**	densely pubescent	glabrous	glabrous
**Disc**	blood red	yellow	yellow-green

### Oreocharis longipedicellata

Taxon classificationPlantaeLamialesGesneriaceae

Lei Cai & F.Wen sp. nov.

DC13DBB6-FA0B-5817-8F97-A5F848EBD40F

urn:lsid:ipni.org:names:77211927-1

Figures 4 , 5

#### Diagnosis.

*Oreocharis longipedicellata* morphologically resembles *O. panzhouensis* Lei Cai, Y.Guo & F.Wen in its ovate leaf blade, yellow flower, four separated fertile stamens, oblong anthers and bilobed, flabellate stigma, but can be easily distinguished from this species in the peduncle 20–28 cm long, bract lanceolate to elliptic, margin denticulate, the calyx 5-lobed to the base, stamens adnate to corolla 3–4 mm from base and the pistil 1.5–2 cm long.

**Figure 4.**
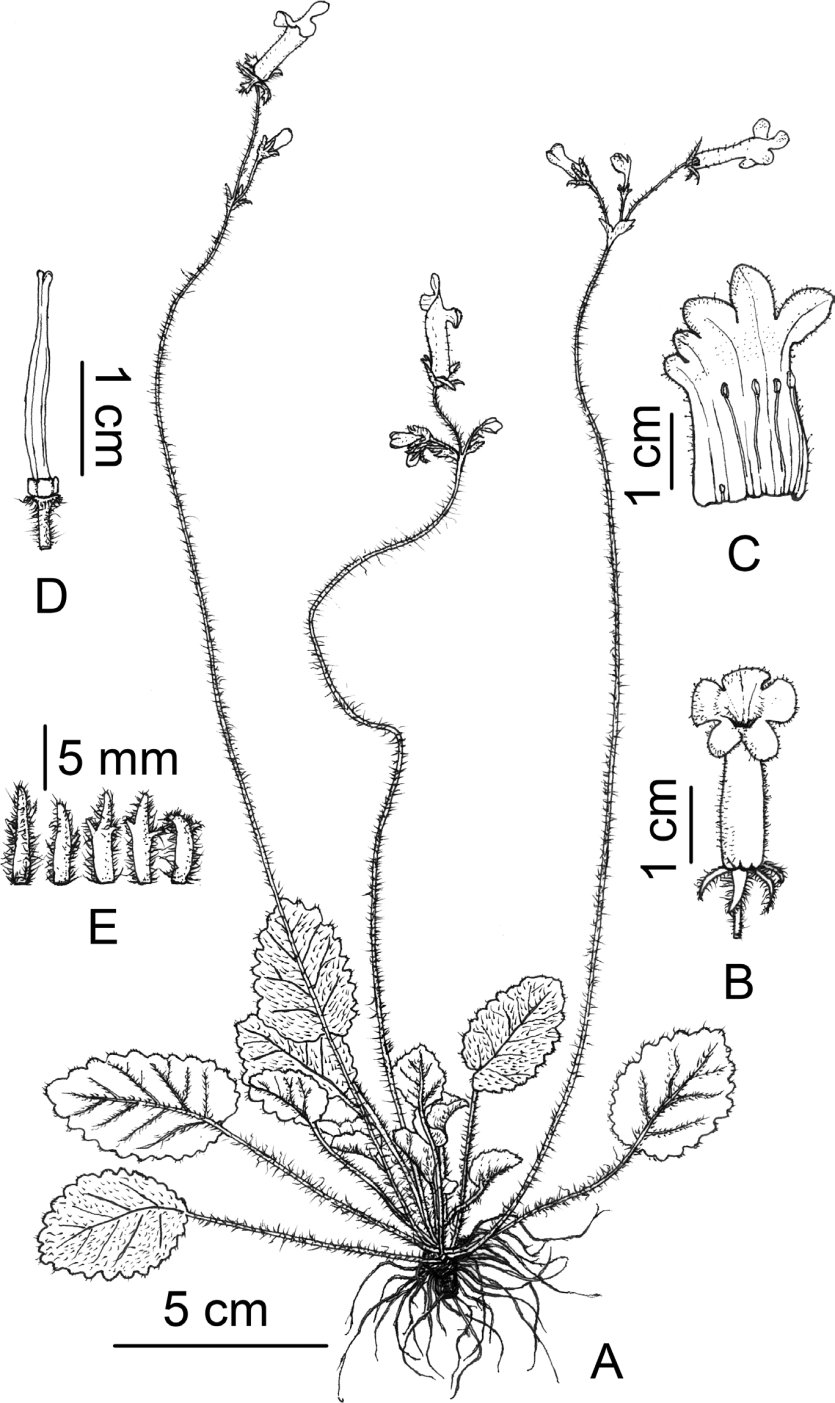
*Oreocharis longipedicellata* Lei Cai & F.Wen, sp. nov. **A** habit **B** front view of flower **C** opened corolla showing stamens and staminode **D** pistil with disc **E** calyx lobes. Drawn by Xuan-Lin Zhu.

#### Type.

China. Yunnan: Malipo County, Mengdong, on the surface of moist rocks (Cultivated in GCCC nursery, Guilin Botanical Garden, Chinese Academy of Sciences) in flower, 24 August 2019, *Fang Wen WF190824-01* (holotype: KUN!, isotype: IBK!).

#### Description.

Perennial herb, rhizome 0.8–2 cm long, 3–5 mm in diameter. Leaves 8–15, basal, petiole 3.5–8.0 cm long, densely brown villous, leaf blade elliptic to ovate, 3.0–5.5 × 2.4–4.5 cm, adaxially densely pubescence, abaxially pubescent, densely brown villous along veins, lateral veins 3–6 on each side of midrib, apex rounded, base slightly oblique sometimes, cordate to auricula-cordate, margin crenate, with brown villous. Cymes axillary 2–5, inflorescence 4–8-flowered; peduncle 20–28 cm long, brown villous; bracts 2, lanceolate to elliptic, 10–12 × 2.5–5.0 mm, adaxially densely villous, abaxially glabrous, sometimes upper part pubescent, margin denticulate; pedicel 2.0–3.5 cm long, densely villous. Calyx 6–9 mm long, 5-lobed to the base, lobes triangular lanceolate to narrowly triangular, 6–9 mm long, 1.5–2 mm wide, outside brown villous, inside glabrous, margin denticulate. Corolla sigmoid, yellow, 2.2–2.8 cm long, outside pubescent and glandular-pubescent, inside glandular-pubescent in the throat and on adaxial lobes, tube cylindrical, slightly bent near the mouth, 1.8–2.2 cm long, 5–7 mm in diameter; limb 2-lipped; adaxial lip 2-lobed to near base, semicircular, lobes 4–5 × 3.8–4.2 mm, abaxial lip 3-lobed to base, semicircular to oval, 6–8 × 5–7 mm. Stamens 4, 1.0–1.3 cm long, adnate to corolla 3–4 mm from the base; filaments linear, glabrous; anthers oblong, 2-loculed, dehiscing longitudinally, connective glabrous; staminode 1, 0.6–1.0 mm long, inserted ca. 1 mm from the base. Disc ca. 1.5 mm high, yellow, margin undulate. Pistil 1.5–2.0 cm long, glabrous; ovary long cylindrical, 10–12 mm long; style 4–6 mm long; stigma bilobed, flabellate. Fruit unknown.

#### Phenology.

Flowering from August to October; fruiting unknown.

#### Distribution and habitat.

*Oreocharis longipedicellata* is currently known by only one population at the type locality, Mengdong, Malipo County, southeastern Yunnan, in the China and Vietnam border area. The species was observed to grow on the surface of moist rocks in the karst region.

#### Etymology.

The specific epithet ‘*longipedicellata*’ refers to the relatively-long peduncle of the new species. This species has almost the longest pedicels in the genus *Oreocharis*.

#### Vernacular name.

The Chinese name of the new species is “Cháng Gěng Mǎ Líng Jù Tái” (长梗马铃苣苔). The first two words, “Cháng Gěng,” mean the long peduncle. The next four words mean *Oreocharis* in mandarin.

**Figure 5.**
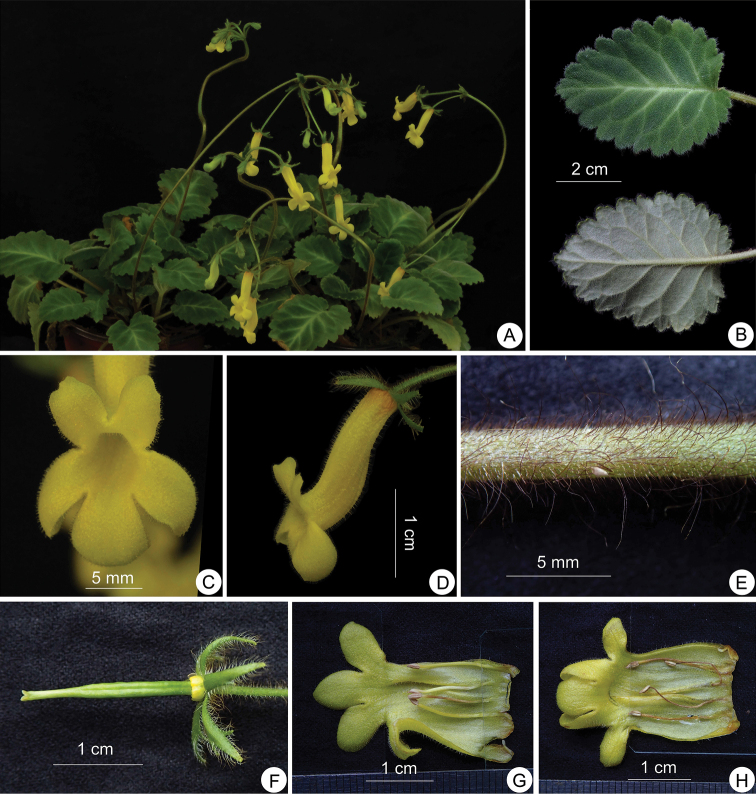
*Oreocharis longipedicellata* Lei Cai & F.Wen, sp. nov. **A** plants cultivated in GBG**B** adaxial and abaxial leaf surface **C** front view of flowers **D** side view of a flower **E** petiole **F** pistil with disc and calyx **G, H** opened corolla showing stamens and staminode. Photographed by Fang Wen.

#### Conservation status.

The new species could be endangered, but more data is needed to evaluate as the field distribution information is not sufficiently detailed.

#### Taxonomic affinities.

*Oreocharis longipedicellata* most resembles recently published *O. panzhouensis* in the yellow flower, four separated stamens, calyx 5-lobed to the middle and stigma bilobed, flabellate. Nevertheless, it differs from the latter species in several other characteristics (see Table 2).

**Table 2.** Morphological comparison between *Oreocharis longipedicellata* sp. nov. and *O. panzhouensis*.

Characters	*O. longipedicellata*	*O. panzhouensis*
**Peduncle**	20–28 cm long	4.5–8 cm long
**Bract**	lanceolate to elliptic, margin denticulate	linear to subulate, margin entire
**Calyx**	5-lobed to the base, lobes lanceolate to narrowly triangular, outside brown villous	5-lobed to the middle, lobes equal, broadly triangular, outside pubescent and sparsely brown villous
**Corolla**	sigmoid, tube cylindrical, lobes reflexed outwards slightly	not sigmoid, tube campanulate, lobes not reflexed outwards
**Stamens**	10–13 mm long, adnate to corolla 3–4 mm from base	5–10 mm long, adnate to corolla 5–6 mm from base
**Pistil**	15–20 mm long, ovary long cylindrical, 10–12 mm long; style 4–6 mm long	8–14 mm long, ovary cylindrical, 5–8 mm long; style 2–4 mm long

## Supplementary Material

XML Treatment for Oreocharis tetraptera

XML Treatment for Oreocharis brachypoda

XML Treatment for Oreocharis aimodisca

XML Treatment for Oreocharis longipedicellata

## References

[B1] Cai L Guo Y Zhang RM Dao ZL Wen F (2019) *Oreocharis panzhouensis* (Gesneriaceae), a new species from karst regions in Guizhou, China. Phytotaxa 393(3): 287–291. 10.11646/phytotaxa.393.3.5

[B2] Cai L Huang H Dao ZL Wu ZK (2017) *Oreocharis parviflora*, a new species of Gesneriaceae from northwestern Yunnan, China. Phytotaxa 329(2): 167–172. 10.11646/phytotaxa.329.2.7

[B3] Chen WH Nguyen QH Chen RZ Nguyen TH Nguyen SK Nguyen VT Möller M Middleton DJ Shui YM (2018) Two new species of *Oreocharis* (Gesneriaceae) from Fan Si Pan, the highest mountain in Vietnam. PhytoKeys 94: 95–106. 10.3897/phytokeys.94.21329 PMC5799783 29416424

[B4] Chen WH Shui YM Möller M (2014) Two new combinations in *Oreocharis* Benth. (Gesneriaceae) from China. Candollea 69(2): 179–182. 10.15553/c2014v692a10

[B5] Do VT Wei YG Wen F (2017) *Oreocharis caobangensis* (Gesneriaceae), a new species from Cao Bang Province, northern Vietnam. Phytotaxa 302(1): 65–70. 10.11646/phytotaxa.302.1.6

[B6] Guo ZY Li ZY Xiang XG (2018) *Oreocharis duyunensis* (Gesneriaceae), a new species from Guizhou, China. Nordic Journal of Botany 36(9): e01514. 10.1111/njb.01514

[B7] IUCN (2019) Guidelines for Using the IUCN Red List Categories and Criteria. Ver. 14. Prepared by the Standards and Petitions Subcommittee of the IUCN Species Survival Commission. http://cmsdocs.s3.amazonaws.com/RedListGuidelines.pdf

[B8] Li JM Li ZM (2015) *Oreocharis brachypodus* (Gesneriaceae), a new taxon from Guizhou, China. Phytotaxa 204(4): 296–299. 10.11646/phytotaxa.204.4.6

[B9] Li ZY Wang YZ (2005) Plants of Gesneriaceae in China. Henan Science & Technology Publishing House, Zhengzhou, Henan, 14–47.

[B10] Middleton DJ Weber A Yao TL Sontag S Möller M (2013) The current status of the species hitherto assigned to *Henckelia* (Gesneriaceae). Edinburgh Journal of Botany 70(3): 385–404. 10.1017/S0960428613000127

[B11] Möller M (2015) Transfer of *Tremacron hongheense* to *Oreocharis* (Gesneriaceae). Phytotaxa 239(3): 295–296. 10.11646/phytotaxa.239.3.12

[B12] Möller M Atkins HJ Bramley GL Middleton DJ Baines R Nguyen VD Bui HQ Barber S (2018) Two new species of *Oreocharis* (Gesneriaceae) from northern Vietnam. Edinburgh Journal of Botany 75(3): 309–319. 10.1017/S0960428618000148

[B13] Möller M Chen WH Shui YM Atkins H Middleton DJ (2014) A new genus of Gesneriaceae in China and the transfer of *Briggsia* species to other genera. Gardens’ Bulletin (Singapore) 66: 195–205.

[B14] Möller M Middleton DJ Nishii K Wei YG Sontag S Weber A (2011) A new delineation for *Oreocharis* incorporating an additional ten genera of Chinese Gesneriaceae. Phytotaxa 23(1): 1–36. 10.11646/phytotaxa.23.1.1

[B15] Möller M Nampy S Janeesha AP Weber A (2017) The Gesneriaceae of India: Consequences of updated generic concepts and new family classification. Rheedea 27(1): 23–41. 10.22244/rheedea.2017.27.1.5

[B16] Pan B Tang GD Do TV Maciejewski S Deng CL Wen F (2019) *Oreocharis tetrapterus* (Gesneriaceae), a new species from East Guangxi, China. PhytoKeys 131: 83–89. 10.3897/phytokeys.131.35434 31576190 PMC6754468

[B17] Wang WT Pan KY Li ZY (1990) Gesneriaceae. In: Wang WT (Ed.) Flora Reipublicae Popularis Sinicae (Vol.69). Science Press, Beijing, 141–271.

[B18] Wang WT Pan KY Li ZY Weitzman AL Skog LE (1998) Gesneriaceae. In: Wu ZY Raven PH (Eds) Flora of China (Vol.18). Science Press, Beijing & Missouri Botanical Garden Press, St. Louis, 254–401.

[B19] Wen F Li S Xin ZB Fu LF Hong X Cai L Qin JQ Pan B Pan FZ Wei YG (2019) The updated plant list of Gesneriaceae in China under the new Chinese naming rules. Guangxi Sciences 26(1): 37–63.

[B20] Wen F Wei YG Fu LF Xin ZB Li S Huang ZJ Meng DC (2014 onward) The Checklist of Gesneriaceae in China. [Free download from] http://gccc.gxib.cn/about-46.aspx

[B21] Xuyen DT Phuong VX Hoan HV Duc NA (2016) Genus *Opithandra* B.L. Burtt and Species *Opithandra dinghushanensis* W.T. Wang as new records for the flora of Vietnam from Bac Huong Hoa Nature Reserve, Quang Tri Province. VNU Journal of Science: Natural Sciences and Technology 32(1S): 142–146.

[B22] Yang LE Cen HF Sun H LoFurno M Maciejewski S Goretsky WJ Wen F (2019) *Oreocharis rubrostriata* (Gesneriaceae), a new species from Guangxi, China. Kew Bulletin 74(23): 1–5. 10.1007/s12225-019-9810-9

